# State Care of Epileptics

**Published:** 1895-12

**Authors:** 


### EDITORIAL DEPARTMENT.

F. E. DANIEL, M. D., Editor.
S. E. HUDSON, M. D., Managing Editor.
A. J. SMITH. M. D., Galveston, Associate Editor.

EDITORIAL STAFF:

PROF. J. E. THOMPSON, M. D., Texas Medical College, Galveston; Surgery.
PROF. WM. KEILLER, M. D., Texas Medical College, Galveston; Obstetrics and Gynecology.
PROF. DAVID CERNA, M. D., Texas Medical College, Galveston; Therapeutics.
PROF. A. J. SMITH, M. D., Texas Medical College, Galveston; Medicine.
DR. R. H. L. BIBB, Saltillo, Mexico; Foreign Correspondent.

Official organ of the West Texas Medical Association, the Houston District Medical Association, the Austin District Medical Society, the Galveston County Medical Society, and several others.

### STATE CARE OF EPILEPTICS.

In furtherance of our advocacy of State care of epileptics, and in the hope of arousing in the minds of Texas physicians an interest in the subject, to the end that it may be brought before the State Association, we reproduce below a portion of an able paper on the subject, from the *Journal of the American Medical Association.* It is by William Francis Drewry, of Petersburg, Va. Dr. Drewry exhausts the subject, leaving little to be said. He opens by directing attention to "the abandoned and neglected class of defectives known as epileptics," and pleads "from the standpoint of physician, humanitarian and political economist for a betterment of their condition." He says epilepsy, to the physician, the statistician, and the socialist, alike, is an unknown and unknownable factor, "as a concomitant of idiocy insanity and crime, this blight upon the human race assumes enormous proportions."

He then discusses the etiology and prognosis of epilepsy, and its relation to insanity; the prevalence of epilepsy; epilepsy in Virginia; the medico-legal aspect of epilepsy, holding with MacDonald that they should be separated from other prisoners, and treated medically. While admitting that "no disease is more intractable to therapeutic measures alone," he says: "Few chronic diseases however, are more amendable to *improvement*, provided judicious treatment, prophylactic, medical and moral—is faithfully carried out."

He comments upon the singularly unhappy lot of the epileptic, and says it is a shame that no discrimination is made in confining them, but that they are thrown in prison with insane, pauper and criminal indiscriminately; speaks of the burden on the family, because of the lack of State care, and says—in italics: "Every principle of humanity and justice revolts against this commingling."

The author continues, coming directly to the subject:

THE STATE THE BEST GUARDIAN OF EPILEPTICS.

What to do for this needy and troublesome class of the population is a question worthy of our deepest consideration. Protection and aid to the victims of the malady, as well as to society in general, demand that provision of some kind be made for their care.

The State government is well adapted to deal with the subject, and should be called upon to take it in hand, and should be urged most earnestly to give it that attention which its importance truly demands.

In the onward march of charity, benevolence and civilization, human suffering of almost every other kind has received governmental aid, and it is a reproach upon this age of progress and enlightenment, that almost nothing has been done to alleviate the pitiable condition of epileptics. It is pleasing, however, to know that in some quarters of the globe interest in them has been awakened, and that this interest is gaining ground.

The question naturally arises, what is the wisest method of giving this much-needed help to epileptics?

INDUSTRIAL COLONY FOR EPILEPTICS.

After years of experience and actual operation, it has become a recognized fact that the special requirements of epileptics are nowhere so well met as in the so-called farm-colony. The prime objects of such a colony are to give each beneficiary the advantages of the most scientific medical treatment, the most humane custodial care, means of regular productive employment and facilities for acquiring an education or a trade. To accomplish these objects, palatial structures are not necessary. Plain, inexpensive pavilions, or cottages, natural and homelike to most of the inmates, shops and other buildings for various industries, a hospital for the sick and the infirm, halls or gymnasiums for recreation and amusement, chapels, schoolhouses, etc., all arranged on the village plan, and attached thereto a large farm, properly equipped, meet the requirements admirably. For those who become insane, isolated buildings of a suitable character should be provided. In such an institution, the beneficiaries would not suffer the ignominy attached to the pauper class, for they would be in a degree, producers and not absolutely dependent.

*　*　*　*　*　*

CARE OF EPILEPTICS IN THIS COUNTRY.

This country, progressive in almost everything and abounding in charity, has been singular slow in recognizing her duty to the thousands of epileptics scattered everywhere throughout our land. True, a few humanitarian associations and legislative enactments have for their object the mitigation of their wretched condition, but there is little tangible proof of our real sympathy for these afflicted and abandoned fellow-creatures.

The first decided step made on this continent, and probably in the world, in the humanitarian care of epileptics, exclusively by the State, was made by Ohio. She opened, in 1893, her hospital for epileptics. The plans adopted contemplate the erection of thirty-six buildings for patients, a number of shops to prove opportunities for the industrial and educational training, as far as practicable, of all the indigent epileptics in the State. In time more land will be purchased in order to enlarge the facilities for employment of the inmates and to supply the institution with all needed vegetables, fruits, etc. Already nine pavilions have been completed and are occupied by 300 patients, whose condition, I am informed by the authorities, has very much improved under the care, proper diet, systematic medical treatment, proper hygienic surroundings, regular exercise, all of which they have the benefit of daily. The medical treatment is conducted by experienced physcians, who are making careful scientific investigations that will in time bear good fruit. The Ohio hospital for epileptics, sane and insane, is a recognized success and blessing.

With high resolve and determined purpose, Drs. Frederick Peterson and W. P. Letchworth, backed up by the State Board of Charities, were instrumental in getting the New York legislature of 1894 to recognize the State's obligation to these unfortunate people. The result was the establishment of the Sonyea colony for epileptics, which with its 1,800 acres of fertile land, abundant water supply, healthy surroundings, cottages workshops, schools, churches, etc., possesses excellent advantages for the proper accommodation of several hundred epileptics. The objects of this great colony are to "provide gratuitously the humane, curative, scientific and economical treatment and care of dependent sane epileptics." It is thought that this institution will in a few years become self-supporting.

The last legislature of Massachusetts, just and generous, authorized the establishment and maintenance at the public expense of a hospital home for epileptics.

The hospital cottage at Baldwinsville, Mass., though a private benevolent institution for non-insane epileptic children, receives a liberal donation from the State.

In California, Pennsylvania, Maryland, Minnesota and Michigan, limited provision, on the industrial and educational plan, has been made at the respective State institutions for the feeble-minded, for the care of sane epileptics. Illinois, Wisconsin, and other States, are being urged by their respective medical societies and boards of charities to erect separate institutions for epileptics.

SUGGESTIONS.

In an article published in the *Virginia Medical Monthly*, September, 1894,—"Care of Epileptics on the Colony Plan,"—I advocated the State care of all dependent epileptics, in an institution exclusively for them. I reiterate the opinion expressed in that paper, viz., that a farm-colony, conducted on the industrial and educational plan and provided with every facility for the most scientific medical treatment of the patients, as sketched in the foregoing pages, would meet the requirements better than any other method yet suggested. *It is feasible, it is economical, it is humane. It has been tried elsewhere, and demonstrated beyond question to be a practical success.*

Who should reap the benefits of such an institution?

1. The epileptics now confined in the hospitals for the insane. By removing these, 200 in number, sufficient room would be gained at the institutions to accommodate probably all of the insane of the State in need of care and treatment, at least for a year or two.

2. All of the epileptics now in the county and city poorhouses—certainly as many as 200.

3. Dependent sane epileptics, each county and city to have at the colony a number of patients proportioned to its population.

4. A limited number of pay patients, probably, who may be in pressing need of special treatment and care.

5. Both white and colored epileptics—separate and distinct provision being made for each race.

To determine the question of epilepsy, proper legal proceedings should be had as in the cases of insanity. The State, however, is sadly in need of a better law regarding the commitment of the insane.

THE DUTY OF PHYSICIANS.

Our lawmakers need to be reminded of the necessity and humaneness of special provision for epileptics. Let us, fellows of the Medical Society of Virginia, with a broad and comprehensive devotion to the general good and to individual happiness, use our best efforts in the creation of a just public sentiment regarding this matter, and, as far as lies in our power, try to "raise the fallen, cheer the faint, and heal the broken-hearted." Public sentiment, the potent power that accomplishes great deeds of mercy and benevolence, never arrays itself against the afflicted and suffering. Only the ignoramus or the narrow-minded bigot will dare discourage any effort to help such unfortunates. If there be any such, tell them the old proverb: "Whoso stoppeth his ears at the cry of the poor, he also shall cry himself, but shall not be heard."

It is not, I believe, Utopian to hope that in the near future our

State will awaken to a recognition of her duty to her epileptics, and see the wisdom of making that provision for them which is clearly demanded by every consideration of justice, equity and philanthropy.

